# Incident Reporting System in an Italian University Hospital: A New Tool for Improving Patient Safety

**DOI:** 10.3390/ijerph17176267

**Published:** 2020-08-28

**Authors:** Davide Ferorelli, Biagio Solarino, Silvia Trotta, Gabriele Mandarelli, Lucia Tattoli, Pasquale Stefanizzi, Francesco Paolo Bianchi, Silvio Tafuri, Fiorenza Zotti, Alessandro Dell’Erba

**Affiliations:** 1 Interdisciplinary Department of Medicine, Section of Legal Medicine, University of Bari, Piazza Giulio Cesare 11, 70100 Bari, Italy; biagio.solarino@uniba.it (B.S.); silvia.trotta89@gmail.com (S.T.); gabriele.mandarelli@uniba.it (G.M.); fiorenzazotti@hotmail.com (F.Z.); alessandro.dellerba@uniba.it (A.D.); 2 Città della Salute e della Scienza di Torino, Turin Hospital, 10126 Torino, Italy; luciatattoli@libero.it; 3 Biomedical Science and Human Oncology, University of Bari, Piazza Giulio Cesare 11, 70100 Bari, Italy; pasquale.stefanizzi@uniba.it (P.S.); frapabi@gmail.com (F.P.B.); silvio.tafuri@uniba.it (S.T.)

**Keywords:** incident reporting, performance management systems, patient safety, clinical risk management, healthcare system resilience, legal medicine

## Abstract

Clinical risk management constitutes a central element in the healthcare systems in relation to the reverberation that it establishes, and as regards the optimization of clinical outcomes for the patient. The starting point for a right clinical risk management is represented by the identification of non-conforming results. The aim of the study is to carry out a systematic analysis of all data received in the first three years of adoption of a reporting system, revealing the strengths and weaknesses. The results emerged showed an increasing trend in the number of total records. Notably, 86.0% of the records came from the medical category. Moreover, 41.0% of the records reported the possible preventive measures that could have averted the event and in 30% of the reports are hints to be put in place to avoid the repetition of the events. The second experimental phase is categorizing the events reported. Implementing the reporting system, it would guarantee a virtuous cycle of learning, training and reallocation of resources. By sensitizing health workers to a correct use of the incident reporting system, it could become a virtuous error learning system. All this would lead to a reduction in litigation and an implementation of the therapeutic doctor–patient alliance.

## 1. Introduction

A right management of clinical risk constitutes, today more than ever, a central element in the healthcare systems in relation to the strong reverberation that it establishes at a socio-cultural, political, organizational, economic level and, above all, as regards the optimization of clinical outcomes due to a non-professional management approach for the patient [1].

The set of actions aimed at identifying the risks to which the patient is submitted/undergone, to quantify their potential effect and to formulate relevant countermeasures; they represent strategic corporate objectives aimed at ensuring the development of a safety culture as a key element to improve the safety of patients and quality of treatment [2]. All this translates into the reduction of the probability that a patient may be the victim of an adverse event, that is, that he suffers any damage or discomfort attributable, even if involuntarily, to the provided medical treatments during the period of hospitalization, which causes an extension of it, a deterioration of health or death [3].

The main objective of clinical risk management systems is represented by the increase in the reliability of health systems, reducing the percentage of predictable adverse events and any mechanism that determines a risk of damage to the patient [4].

In a systemic and systematic perspective of a right clinical risk management, different methods, tools and actions are expected to revel risk sources, to provide useful evidences about the incident of adverse events, and therefore to reach a reduction and containment of the same [5,6]. These processes take advantage of proactive precise tools that hospitals experiment and use with different degrees of awareness, the main of which are safety briefings, focus groups, analysis and review of medical records, screening, observations, patient safety walkaround and global trigger tool [7].

Once the system risks and deficiencies have been identified using these tools, the second application phase involves the use of additional clinical risk management techniques (FMEA/FMECA; root cause analysis; clinical audits; etc.) aimed at preventing the planning of certain suitable protective barriers and the identification of the priorities order in relation to the interventions to implement [8].

The starting point for a right management of the hospital’s clinical risk is therefore represented by the identification and analysis of non- conforming results, determining whether they have caused damage to the patient [9]. A useful tool for this is represented by incident reporting system [10].

Leading patient safety experts acknowledge the current challenges of incident reports. The future of incident reporting lies in targeted incident reporting, effective triaging and robust analysis of the incident reports and meaningful engagement of doctors [11]. Incident reporting must be coupled with visible, sustainable action, because the way in which the medical profession reports serious and other incidents still needs to be improved [12].

The use and the operation in healthcare systems of incident reporting systems have been extensively studied in the literature. Cuong Pam et al. explained that those systems are, and will continue to be, an important influence on improving patient safety, by providing valuable insights into how and why patients can be harmed at the organizational level. However, they have several limitations that should be considered as inherent biases of voluntary reporting [13].

## 2. Materials and Methods

By accessing the computerized incident reporting system of the “Policlinico di Bari” academic hospital (>1500 beds; >50,000 hospitalizations/year), the aim of the study is to carry out a systematic analysis of all data received by the Clinical Risk and Patient Safety Management Unit in the first three years of adoption of a reporting system by the hospital (period 2015–2018).

This analysis reveals the strengths and weaknesses of the hospital systems currently in use in Italian hospitals; to outline trends of acceptance of the tool by operators; to identify factors that determine the correct/incorrect, complete/incomplete compilation and transmission with reference to each single operating unit; to analyse result indicators in terms of implementing the culture of learning the error and the lack of awareness among health workers [11].

At the same time, through the stratification of the reported events in the sheets, the aim is to suggest any structural changes to the tool, in order to increase the operator’s acceptance to the use of the incident reporting systems, by channelling the information flows with a view of the training implementation of the operators themselves [12].

Overall, 200 incident reporting forms were analysed, transmitted via the computer system of the University Hospital—Policlinico of Bari to the Clinical Risk Management and Patient Safety Unit, in the period November 2015–November 2018, i.e., from the beginning of the adoption of this corporate reporting system.

For each received individual reporting form, the general data relating to the patient’s personal data section (Surname and Name; Date of birth/Age; Number of hospitalization) were collected and analysed, with the aim of assessing the possible incidence of events in relation to the age of the patients.

Therefore, the analysis was then carried out, for each individual case, of the operating unit in which the occurred event, the general information and the qualification (Doctor; Nurse; Other) of the editor.

With regard to the “Type of service”, we proceeded with the stratification of the patients’ reasons for accessing the Bari Teaching Hospital, distinguishing them between ordinary hospitalizations, home services, surgical interventions, outpatient services, day hospital admissions and other reasons for access.

Following the structure of the computer form, we continued with the analysis and definition of the events that occurred, with the aim of both categorizing adverse events, near misses and sentinel events, and to evaluate, case-by-case, if we determined the occurrence of a delay in the procedure to be delivered, of an incorrect or inappropriate procedure, of a failed procedure or of other events, which in any case represented an adverse event.

As regards the “Delay in the procedure”, it was assessed whether it had concerned a diagnostic procedure, a therapeutic procedure, an assistance service, a surgical procedure, a drug administration or a rehabilitation service [14].

In relation to the “inappropriate procedures”, it was assessed whether they had been determined by an incorrect identification of the patient/side; incorrect administration of the drug; inappropriate rehabilitation service; incorrect surgical procedure; inappropriate diagnostic procedure; inappropriate therapeutic procedure; incorrect therapeutic procedure; inappropriate surgical performance.

In the case of “Failure to proceed”, the nature of this omission was determined, and whether it concerned a diagnostic procedure, a therapeutic procedure, an assistance service, a surgical procedure or an incorrect administration of drugs.

In the “Other” section, there are other entries, not included in the fields previously defined, which constitute adverse events and potentially the cause of sentinel events, in the case represented by aggression to operator; patient transport; accidental fall; blood components and/or blood products transfusion; use of implants/equipment; service-related infections; the new onset of pressure injuries; dangerous environmental conditions; other.

The incident reporting form, after these pre-filled fields with the possibility of individual choice by the operator, it provides a “narrative” section relating to the “Description of events—Conduct of the facts”, in which the operator can, within a free field, describe in more detail “What happened”; “Where”, “When”, “How and why it happened”. An analysis was therefore conducted, always for each individual case of these items, in relation to date, time, place, day and description of the events.

After these first fields relating to the actual “reporting” of the events, we proceeded with the study of the sections based on “learning”, and on the experiential observation made by the operators.

The form provides for the identification of the “Factors that may have contributed to the event”. These factors, subject to further systematic analysis, may be related to the patient, staff or system.

Once we analysed any contributing factors, identified by the operators themselves, we continued with the analysis of any “Factors that may have reduced the outcome”, such as early detection, randomness, compliance with protocols/procedures, or other factors.

The last section of the form focuses on what the occurrence of the event could have determined in terms of “Further investigations or performances”, such as instrumental or laboratory investigations; admissions; surgical interventions; medications or specialist advice.

Having examined these additional data, the last analysis was carried out on the eventual documentation of the events that took place in the medical record, on the relative information to the patient, on “how the event could be prevented/avoided”, and on the level of severity reverberated towards the patient (no damage; mild; medium; severe; death).

**Figure 1:**
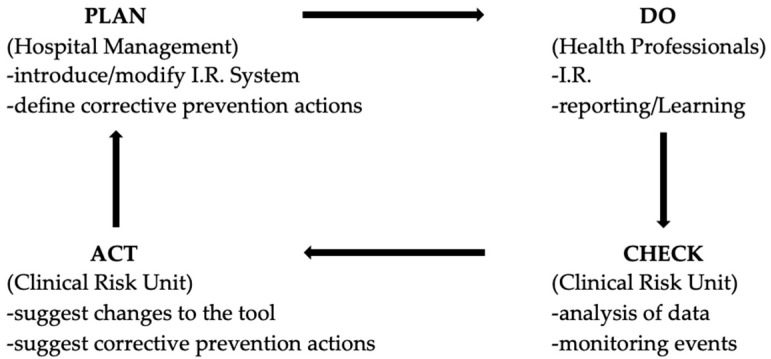
Clinical Risk Management Cycle.

With this analysis it was possible to demonstrate that the information arrived through the correct compilation of the incident reporting form by the operator is the starting point, for the clinical risk units, to be able to introduce a corrective prevention action. The feedback of action and the continuous monitoring of the events will ensure greater patient safety and will guarantee a virtuous learning system for all involved, according to the Deming cycle, and ensuring the continuous improvement of healthcare (Figure 1) [15].

The study model is of a transversal observational type.

The extracted reports were entered on a database built using Office Excel software (Microsoft Corporation One Microsoft Way Redmond, WA 98052-6399, USA), and analysed through Stata MP15 software (StataCorp LLC 4905 Lakeway Drive College Station, TX 77845-4512, USA).

Continuous variables were expressed as mean ± standard deviation and range, categorical variables as proportions.

Univariate logistic regression was used to evaluate the association between the reports of serious event (including death) and the age of the patient, the type of performance, the presence of the event in the medical record, information of the patient and the year of the event; the odds ratio was calculated, with an indication of the 95% confidence interval (95% CI) and the z-score test. It was not possible to build a multivariate logistic regression model, as no determinant seems to be associated with the univariate outcome.

For all tests, a value of *p* < 0.05 was considered significant.

## 3. Results

This section may be divided by subheadings. It should provide a concise and precise description of the experimental results, their interpretation, as well as the experimental conclusions that can be drawn.

The study sample consists of 200 Incident Reporting forms; over the years under analysis, an increasing trend in the number of total reports was demonstrated (2015: 13 reports; 2016: 34 reports; 2017: 69 reports; 2018: 84 reports), indicating a greater use of the tool by healthcare professionals.

The greatest proportion of reports was sent by the Psychiatric Operating Unit (*n* = 58/200; 29.0%); in the period under analysis for some operating units, there was an increase in reports over the years (Psychiatry and First Aid); for others there was a decreasing trend (Pediatrics and Gastroenterology) (Table 1).

The professional figure that has made the most frequent records is that of the Doctor (*n* = 172/200; 86.0%), followed by the Nurse (*n* = 26/200; 13.0%) and by another Healthcare Professional (*n* = 2/200; 1.0%); this distribution has remained constant over the years under analysis.

The age of the subjects affected by the events reported was known for 155/200 (77.5%) patients, who have an average age of 46.0 ± 28.4 years (range = 1.0–94, 0). The average age showed a growing trend in the period under analysis (2015: 28.1 ± 31.1; range = 1.0–83.0; 2016: 35.7 ± 30.5; range = 1.0–87.0; 2017: 45.6 ± 28.4; range = 1.0–92.0; 2018 = 55.4 ± 23.4; range = 1.0–94.0).

The type of service was identified in 181/200 (90.5%) reports, with 129/181 (71.3%) events that occurred in the ordinary regime, 16/181 (8.8%) in the outpatient regime, 4/181 (2.2%) in the surgical procedure, and 32/181 (17.7%) in the other regime; it has been observed that over the years, reports in the ordinary regime have increased, and those in the outpatient regime have decreased.

With regard to the categorization of events, healthcare professionals reported: 1/200 (0.5%) event due to delay in the procedure (drug reporting); 15/200 (7.5%) events due to incorrect/inappropriate procedure of which: 6/15 (40.0%) incorrect surgical procedure, 3/15 (20.0%) incorrect patient identification, 2/15 (13.3%) inappropriate surgical performance, 2/15 (13.3%) inappropriate diagnostic procedure, 1/15 (6.7%) inappropriate therapeutic procedure, 1/15 (6.7%) incorrect therapeutic procedure; 8/200 (4.0%) events due to non-procedure, of which 3/8 (37.5%) diagnostic procedure, 2/8 (25.0%) assistance, 1/8 (12.5%) incorrect drug administration, 1/8 (12.5%) surgical performance, 1/8 (12.5%) therapeutic procedure; 177/200 (88.5%) events due to “others”, of which 84/177 (47.5%) accidental fall, 52/177 (29.4%) operator aggression, 19/177 (10.7%) others, 7/177 (4.0%) care-related infections, 6/177 (3.4%) environmental conditions, 4/177 (2.3%) transportation, 3/177 (1.7%) use of implants/equipment, 1/177 (0.5%) transfusion, 1/177 (0.5%) patient aggression.

For a report, there were multiple motivations. From the analysis of motivated records as “others” in the period under analysis, it has been observed that, over the years, reports of aggression against operators have increased, and those related to accidental falls have decreased (Table 2).

The cause of the event reported with the greatest prevalence is represented by the falls (*n* = 85/200; 41.5%; graph 3); a decreasing trend was observed as the cause of the event, while physical attacks were increasing (Table 3).

For 197/200 (98.5%) reports, the place where the event took place was known, and the hospitalization ward was the most frequently reported setting (*n* = 82/197; 41.6%); an increasing trend was observed for ward accidents, while those that occurred in the operating room seemed to have decreased (Table 4).

For 196/200 (98.0%) reports, the phase of the day in which the event took place was known; the night emerged as the most frequently reported phase (*n* = 72/196; 36.7%) (Table 5).

Most records reported events that occurred on weekdays (*n* = 171/200; 85.5%), without significant differences in the distribution by type of day (weekdays/holidays) in the years under analysis (2015 = 11/13; 84.6%; 2016 = 28/34; 82.4%; 2017 = 60/69; 87.0%; 2018 = 72/84; 85.7%).

Notably, 99/200 (49.5%) records reported patient-related factors that would have favored the event; of these, the patient’s general condition represented the most frequent finding (*n* = 77/99; 77.8%; graph 4); this feedback has remained constant over the years under analysis.

Moreover, 28/200 (14.0%) records reported personnel-related factors that would have favored the event, with the stress/fatigue of healthcare workers being the most frequent factor (*n* = 17/28; 60.7%; graph 5); this feedback has remained constant over the years under analysis.

Moreover, 61/200 (30.5%) records reported factors related to the system that would have favored the event, with inadequate staff representing the most frequent factor (*n* = 21/61; 34.4%; graph 6); this feedback has remained constant over the years under analysis.

Moreover, 17/200 (8.5%) records reported other factors that would have favored the event, with the distraction of relatives being the most frequent factor (*n* = 5/17; 29.4%); this feedback has remained constant over the years under analysis.

Moreover, 118/200 (59.0%) records reported the preventive measures that made it possible not to increase the severity of the event, with early detection which represented the most frequently encountered factor (*n* = 88/118; 74.6%); this feedback has remained constant over the years under analysis.

Moreover, 147/200 (73.5%) records reported investigations required following the event, with the most frequently applied instrumental investigations (*n* = 62/147; 42.2%); this feedback has remained constant over the years under analysis.

Moreover, 83.0% (*n* = 166/200) of the records reported that the event was marked in the medical chart; this correct procedure tends to be less and less respected in the years under analysis (2015 = 12/13; 92.3%; 2016 = 29/34; 85.3%; 2017 = 58/69; 84.1%; 2018 = 67/84; 79.8%).

Moreover, 85.0% (*n* = 170/200) of the records reported that the patient had been informed of the event; this correct procedure tends to be less and less respected in the years under analysis (2015 = 13/13; 100.0%; 2016 = 30/34; 88.2%; 2017 = 56/69; 81.2%; 2018 = 71/84; 84.5%).

Moreover, 82/200 (41.0%) records reported a possible preventive measure that could have averted the event; the greater proportion of whistle-blowers reported that it would have been impossible to prevent the event (*n* = 22/82; 26.8%) or that it would have been possible to prevent it with better education/training of health staff (*n* = 22/82; 26.8%).

For 199/200 (99.5%) records, the severity of the event was disclosed, with 2/199 (1.0%) deaths, 13/199 (6.5%) serious events, 37/199 (18.6%) medium, 72/199 (36.2%) mild and 75/199 (37.7%) no damage; in the period under analysis, there were no significant differences in the severity of events per year (Table 6).

To understand the improvement measures to be implemented to reduce near-misses and adverse events, an analysis of the factors that, according to health professionals, favored the adverse event has been carried out.

Notably, 99/200 (49.5%) records reported patient-related factors that would have favored the adverse event; of these, the patient’s general condition represents the most frequent factor (*n* = 77/99; 77.8%).

Moreover, 28/200 (14.0%) records reported staff-related factors. Stress and fatigue of operators represent the most frequent staff-related factor (*n* = 17/28; 60.7%).

Finally, 61/200 records reported system-related factors. Healthcare professional’s inadequacy represent the most frequent staff-related factor (*n* = 21/61; 34.4%).

These findings have been constant in the years analyzed.

No statistically significant associations were observed between the reporting of a serious event and the determinants under analysis (*p* > 0.05).

## 4. Discussion

Having set the starting point for the right management of the hospital’s clinical risk in the identification and analysis of the results that do not meet expectations, the information flows emerged from the incident reporting system adopted in the University Hospital—Policlinico Hospital of Bari, with the aim of carrying out a systematic analysis of all data received by the Patient’s Clinical Risk and Safety Management Unit, through the incident reporting system, in the period 2015–2018, i.e., starting from the date of adoption, by the hospital, of the reporting system. This is an important strength of the article because, unlike the others already present in the literature, an analysis of the data was carried out, starting from the introduction of the incident reporting system.

The results emerged from the analysis of the 200 forms, showing a general increasing trend in the number of total records, indicating both a greater use of the tool by healthcare professionals, and the positive repercussions that the dissemination and awareness of the use of the tools reporting conditions have a health level [16]. The number of reports is a weakness, but this is normal when introducing a new clinical risk management tool in a hospital (Table 7).

Furthermore, 86.0% of the records came from operators belonging to the medical category, 13.0% from nurses and 1.0% from other health professionals. The reason for this discrepancy can certainly be traced back to the responsibility of the various professional figures, with the category of doctors most interested in reporting for possible legal implications [17]. What is certain is that, although continuing to work on the figure of doctors, a necessary awareness-raising work must be completed on the other professional categories, both to achieve a banal increase in records, and, above all, to be able to grasp important ideas on the measures of risk containment to be put in place, including by nursing and other health professionals who, remaining in close contact with users, could provide valuable stimuli and elements to be elaborated on, in order to reach risk containment systems for patients.

A further datum is the imbalance in the use of the incident reporting tool with “reporting” in the strict sense, compared to “learning”.

From the analysis carried out, it emerged that only 41.0% (82/200) of the records reported the possible preventive measures that could have averted the event and, of these, 26.8% (22/82) concern the impossibility of being able to prevent the event itself. Therefore, only in 30% of the reports are hints and suggestions from those directly concerned regarding the potential restraint measures to be put in place to avoid the repetition of the events, thus precluding, for more than 2/3 of the reports, the possibility of seizing ideas for practical intervention, and to carry out sustainable learning with the operators [18,19]—so much more important in the consideration of the further relevant data that emerged during the analysis, or that relating to the investigations that became necessary following the event that occurred. In 73.5% of records (147/200), investigations required following the occurrence of events were reported, with the most frequently applied instrumental investigations (42.2% of cases). These assessments, as easily understood, determine an important investment of human and economic resources, precluding the possibility of being able to allocate them elsewhere [20].

Moving on to the second experimental phase of our study, or the categorization of the events reported with the intent of being able to circumscribe and identify the most frequent events, in order to achieve interventions aimed at containing risks that they may recur, we remind that, using the macro areas provided from the incident reporting data sheet, health professionals reported 0.5% of events due to a delay in the procedure; 7.5% of events due to incorrect/inappropriate procedure; 4.0% of events due to non-procedure; 88.5% of events due to other reason. In relation to this categorization, there is immediately a substantial difficulty in being able to categorize the events reported in macro-areas of intervention [21].

By studying the results that emerged from the analysis of the incident reporting system, the hospital’s Clinical Risk and Safety Management Unit identified the areas of intervention and the risk management tools, completing the Clinical Risk Management Cycle.

First of all, numerous meetings were held with the staff of the various hospital departments, to sensitize health professionals to the use of the incident reporting system. During these meetings, the focus was mainly on nurses and other health professionals, in light of the low number of reports received from these professional categories.

In addition, several measures were performed in the different hospital departments with the greatest number of reports. Patient safety walkarounds [7], global trigger tool analysis, quantification and causes analysis of overprescription [22], study of determinants in deaths [23], adoption and implementation of Surgical Safety Checklist and Handover Checklist [8,9,10,11,12,13,14,15,16,17,18,19,20,21,22,23,24], and projects for the reduction of hospital infection were all performed [14].

Thanks to these improvement measures, it is hoped, in accordance with international scientific literature, that there will be a decrease in near misses and adverse events. For this reason, an analysis of the results which emerged from the incident reporting system will be done every three years.

## 5. Conclusions

A systematic analysis of all data received by the Clinical Risk and Patient Safety Management Unit in the first three years of adoption of a reporting system has been carried out. The not high number of reports is a function of introduction of a new clinical risk management tool in an academic hospital, and this will allow the usage of tool monitoring.

Despite this aspect, the important fact is that our analysis is about the first three years since the adoption of the system. Therefore, it was possible to make an assessment of the impact of the incident reporting system introduction, and to evaluate, by monitoring the first period of adoption, the increasing use by health professionals, the critical tool’s issues, and the improvement of the learning process.

Implementing the reporting system, and sections concerning possible preventive measures that could prevent the repetition of the event in the future, it would guarantee a virtuous cycle of learning, training and reallocation of resources, which, in practice, would translate into transformation of the inputs received directly from the operators into procedures and protocols which, once they are introduced and interjected by the operators, it would guarantee a certain reduction of unwanted events, having, as a result, both a direct greater safety of the treatments and an indirect possibility of being able to carry out training on health workers, with a guarantee of saving of resources made necessary due to the occurrence of events, and with the possibility of a structural reallocation of the resources themselves [25].

Some structural changes to the incident reporting forms are required, represented by the reduction of the free text entry fields in favor of fixed menus relating to the specific categories of adverse event, allowing operators to perform faster, useful and complete compilation of the incident reporting forms.

Furthermore, it would be appropriate to create a more widespread categorization system of events, identifying specific areas for intervention and improvement [26].

By implementing and sensitizing health workers, belonging to the various job categories, to a correct use of the incident reporting tool, not only in the portion relating to the reporting of the event, but also, and above all, in the sections related to learning, it could become a virtuous error learning system [27].

This would allow the Clinical Risk Management Units to receive reports of any adverse events, to identify factors that led to their occurrence, as well as the specific critical areas of intervention.

Through the development and implementation of protocols, procedures and good care practices suggested, always through the incident reporting forms, directly by the health workers involved, it would be possible, in practice, to make certain protective barriers towards patients who, contextually, would represent analytical process indicators, relative to the implementation of the quality of care [28,29].

All this would reverberate positively on the healthcare systems, both in terms of involvement of the operators within the care and decision-making processes, and in terms of implementation of the quality and safety levels of the treatments, leading to a reduction in litigation, an important reduction in the frequency of patient safety incidents, and an implementation of the therapeutic doctor–patient alliance, with a view of modern medicine that is less and less “defensive” and more and more “no blame” [30,31].

It will be interesting and necessary to also continue the analysis of the data in the future, to see if this will be the case.

## Figures and Tables

**Table 1:** Distribution (%) of Incident Reporting Records, by Complex Operating Unit and the reporting year.

O.U.	2015 (*n* = 13)	2016 (*n* = 34)	2017 (*n* = 69)	2018 (*n* = 84)
	*n*. (%)	*n*. (%)	*n*. (%)	*n*. (%)
Psychiatry	1 (7, 7)	6 (17, 7)	22 (31, 9)	29 (34, 5)
First Aid	0	3 (8, 8)	13 (18, 8)	16 (19, 1)
Pediatrics	6 (46, 2)	9 (26, 5)	10 (14, 5)	6 (7, 1)
Neurosurgery	0	1 (2, 9)	4 (5, 8)	14 (16, 7)
Gastroenterology	2 (15, 3)	5 (14, 7)	3 (4, 4)	1 (1, 2)
Ophthalmology	3 (23, 1)	4 (11, 8)	1 (1, 5)	0
Internal Medicine	0	0	4 (5, 8)	1 (1, 2)
Orthopedics	0	0	0	5 (6)
Otolaryngology	0	0	4 (5, 8)	1 (1, 2)
Nephrology	0	1 (2, 9)	2 (2, 9)	0
Others O.U.	1 (7, 7)	3 (8, 8)	8 (11, 4)	10 (11, 8)

**Table 2:** Distribution (%) of Incident Reporting records motivated as “others”, the reporting year.

Incident Reporting	2015 (*n* = 12)	2016 (*n* = 31)	2017 (*n* = 59)	2018 (*n* = 75)
	*n*. (%)	*n*. (%)	*n*. (%)	*n*. (%)
Accidental fall	9 (75)	21 (67, 7)	25 (42, 4)	29 (38, 7)
Operator aggression	0	6 (19, 4)	21 (35, 6)	25 (33, 3)
Others	2 (16, 7)	1 (3, 2)	6 (10, 2)	10 (13, 3)
ICA	0	0	1 (1, 7)	6 (8)
Environmental cond.	1 (8, 3)	2 (6, 5)	2 (3, 4)	1 (1, 3)
Transport	0	0	3 (5, 1)	1 (1, 3)
Use of impl./equipm.	0	0	1 (1, 7)	2 (2, 8)
Transfusion	0	1 (3, 2)	0	0
Patient aggression	0	0	0	1 (1, 3)

**Table 3:** Distribution (%) of Incident Reporting records, by reason of event and the reporting year.

Cause	2015 (*n* = 13)	2016 (*n* = 34)	2017 (*n* = 69)	2018 (*n* = 84)
	*n*. (%)	*n*. (%)	*n*. (%)	*n*. (%)
Fall	9 (69, 2)	21 (61, 8)	26 (37, 7)	29 (34, 5)
Physical aggression	0	3 (8, 8)	12 (17, 4)	18 (21, 4)
Verbal Aggression	0	3 (8, 8)	9 (13)	7 (8, 3)
Aggr. between patients	0	0	2 (2, 9)	5 (6)
Surgical Site Infections	0	0	1 (1, 4)	6 (7, 1)
Environmental damage	0	0	2 (2, 9)	2 (2, 4)
Trauma	0	2 (5, 9)	1 (1, 4)	1 (1, 2)
Others	9 (69, 2)	21 (61, 8)	26 (37, 7)	29 (34, 5)

**Table 4:** Distribution (%) of Incident Reporting records, by place of event and the reporting year.

Place	2015 (*n* = 13)	2016 (*n* = 34)	2017 (*n* = 69)	2018 (*n* = 84)
	*n*. (%)	*n*. (%)	*n*. (%)	*n*. (%)
Ward	4 (30, 8)	7 (21, 9)	25 (36, 8)	46 (54, 8)
Room	5 (38, 4)	11 (34, 4)	16 (23, 5)	28 (33, 3)
Operating Room	2 (15, 4)	4 (12, 5)	10 (14, 7)	6 (7, 1)
Toilet	0	5 (15, 6)	4 (5, 9)	1 (1, 2)
Others	2 (15, 4)	5 (15, 6)	13 (19, 1)	3 (3, 6)

**Table 5:** Distribution (%) of Incident Reporting records, by phase of the day and the reporting year.

Phase of the Day	2015 (*n* = 13)	2016 (*n* = 34)	2017 (*n* = 69)	2018 (*n* = 84)
	*n*. (%)	*n*. (%)	*n*. (%)	*n*. (%)
Night	3 (23,1)	15 (44,1)	22 (32,3)	32 (39,5)
Morning	8 (61,5)	10 (29,4)	25 (36,8)	28 (34,6)
Afternoon	2 (15,4)	9 (26,5)	21 (30,9)	53 (27,1)

**Table 6:** Distribution (%) of Incident Reporting records that report the severity of the event (*n* = 199), by severity and year of reporting.

Severity of Event	2015 (*n* = 13)	2016 (*n* = 34)	2017 (*n* = 69)	2018 (*n* = 84)
	*n*. (%)	*n*. (%)	*n*. (%)	*n*. (%)
No damage	6 (46, 2)	14 (41, 2)	24 (35, 3)	31 (36, 9)
Mild	3 (23, 1)	16 (47, 1)	29 (42, 6)	24 (28, 6)
Middle	3 (23, 1)	4 (11, 7)	9 (13, 2)	21 (25)
Serious	1 (7, 6)	0	5 (7, 4)	7 (8, 3)
Death	0	0	1 (1, 5)	6 (1, 2)

**Table 7:** Main strengths and weaknesses of the introduction of the incident reporting system.

Strengths	Weaknesses
Category of doctor is very interested.	Limited number of IRS. Other professional categories need awareness-raising actions.
Analysis also allows to identify waste of resources.	In few reports are reported ideas for practical intervention.
High impact on learning.	Difficulty to categorize the events in macro-areas of intervention.

## References

[1] Rodriguez A.L., Zupancic S., Song M.M., Cordero J., Nguyen T.Q., Seifert C. (2017). Importance of an Interprofessional Team Approach in Achieving Improved Management of the Dizzy Patient. J. Am. Acad. Audiol..

[2] Weaver S.J., Lubomsky L.H., Wilson R.F., Pfoh E.R., Martinez K.A., Dy S.M. (2013). Promoting a culture of safety as a patient safety strategy: A systematic review. Ann. Intern. Med..

[3] Els C., Jackson T.D., Kunyk D., Lappi V.G., Sonnenberg B., Hagtvedt R., Sharma S., Kolahdooz F., Straube S. (2017). Adverse events associated with medium- and long-term use of opioids for chronic non-cancer pain: An overview of Cochrane Reviews. Cochrane Database Syst. Rev..

[4] King A., Bottle A., Faiz O., Aylin P. (2017). Investigating Adverse Event Free Admissions in Medicare Inpatients as a Patient Safety Indicator. Ann. Surg..

[5] Gorrel L.M., Engel R.M., Lystad R.P., Brown B.T. (2017). Assignment of adverse event indexing terms in randomized clinical trials involving spinal manipulative therapy: An audit of records in MEDLINE and EMBASE databases. BMC Med. Res. Methodol..

[6] Klein D.O., Rennenberg R.J.M.W., Koopmans R.P., Prins M.H. (2018). Adverse event detection by medical record review is reproducible, but the assessment of their preventability is not. PLoS ONE.

[7] Ferorelli D., Zotti F., Tafuri S., Pezzolla A., Dell’Erba A. (2016). Patient Safety Walkaround: A communication tool for the reallocation of health service resources: An Italian experience of safety healthcare implementation. Medicine.

[8] Ferorelli D., Giandola T., Laterza M., Solarino B., Pezzolla A., Zotti F., Dell’Erba A. (2017). Handover checklist: Testing a standardization process in an Italian hospital. Risk Manag. Health Policy.

[9] Wiles R., Cott C., Gibson B.E. (2008). Hope, expectations and recovery from illness: A narrative synthesis of qualitative research. J. Adv. Nurs..

[10] Leistikow I., Mulder S., Vesseur J., Robben P. (2017). Learning from incidents in healthcare: The journey, not the arrival, matters. BMJ Qual. Saf..

[11] Mitchell I., Schuster A., Smith K., Pronovost P., Wu A. (2016). Patient safety incident reporting: A qualitative study of thoughts and perceptions of experts 15 years after ‘To Err is Human’. BMJ Qual. Saf..

[12] Archer G., Colhoun A. (2018). Incident reporting behaviours following the Francis report: A cross-sectional survey. J. Eval. Clin. Pract..

[13] Cuong Pham J., Girard T., Pronovost P.J. (2013). What to do With Healthcare Incident Reporting Systems. J. Public Health Res..

[14] Ferorelli D., Zotti F., Tafuri S., Pezzolla A., Dalfino L., Brienza N., Dell’Erba A. (2015). Good medical practices in the use of antibiotic prophylaxis in a surgery ward: Results of a 2013 Apulian study. Am. J. Infect. Control.

[15] Harolds J. (2015). Quality and Safety in Health Care, Part I: Five Pioneers in Quality. Clin. Nucl. Med..

[16] Tricarico P., Castriotta L., Battistella C., Bellomo F., Cattani G., Grillone L., Degan S., De Corti D., Brusaferro S. (2017). Professional attitudes toward incident reporting: Can we measure and compare improvements in patient safety culture?. Int. J. Qual. Health Care.

[17] Thoma J.E., Waite M.A. (2018). Experiences of nurse case managers within a central discharge planning role of collaboration between physicians, patients and other healthcare professionals: A sociocultural qualitative study. J. Clin. Nurs..

[18] McEvoy F.J., Shen N.W., Nielsen D.H., Buelund L.E., Holm P. (2017). Online Radiology Reporting with Peer Review as a Learning and Feedback Tool in Radiology; Implementation, Validity, and Student Impressions. J. Digit. Imaging.

[19] Probst T.M., Petitta L., Barbaranelli C. (2017). Comparing recall vs. recognition measures of accident under-reporting: A two-country examination. Accid. Anal. Prev..

[20] Mizban L., El-Belihy M., Vaidyanathan M., Brown J. (2019). An audit and service evaluation of the use of cone beam computed tomography (CBCT) in a paediatric dentistry department. Dentomaxillofac. Radiol..

[21] Brunsveld-Reinders A.H., Arbous M.S., De Vos R., De Jonge E. (2016). Incident and error reporting systems in intensive care: A systematic review of the literature. Int. J. Qual. Health Care.

[22] Ferorelli D., Donno F., De Giorgio G., Mele F., Favia M., Riefoli F., Andresciani S., Melodia R., Zotti F., Dell’Erba A. (2020). Head CT scan in emergency room: Is it still abused? Quantification and causes analysis of overprescription in an Italian Emergency Department. Radiol. Med..

[23] Ferorelli D., Donno F., De Giorgio G., Zotti F., Dell’Erba A. (2020). Study of determinants in deaths occurring in an Italian teaching hospital during a year. Clin. Ter..

[24] Ferorelli D., Crudele L., Vicenti L., Zotti F., Dell’Erba A. (2019). Adoption and Implementation of the Surgical Safety Checklist: Improving Safety in an Italian Teaching Hospital. Am. J. Med. Qual..

[25] Massey D., Aitken L.M., Chaboyer W. (2015). The impact of a nurse led rapid response system on adverse, major adverse events and activation of the medical emergency team. Intensive Crit. Care Nurs..

[26] Hohenstein C., Fleischmann T., Rupp P., Hempel D., Wilk S., Winning J. (2016). German critical incident reporting system database of prehospital emergency medicine: Analysis of reported communication and medication errors between 2005–2015. World J. Emerg. Med..

[27] Metcalfe J. (2017). Learning from Errors. Annu. Rev. Psycol..

[28] Di Mare V., Garramone G., Rubbiani M., Moretto A. (2017). Quality check of safety data sheets for plant protection product co-formulants: Hazard classification and coherence of the information. Med. Lav..

[29] Metcalfe J., Xu J. (2018). Learning from one’s own errors and those of others. Psychon. Bull. Rev..

[30] Ferguson C.C. (2017). The Emotional Fallout from the Culture of Blame and Shame. JAMA Pediatr..

[31] Ramírez E., Martín A., Villán Y., Lorente M., Ojeda J., Moro M., Vara C., Avenza M., Domingo M.J., Alonso P. (2018). Effectiveness and limitations of an incident-reporting system analyzed by local clinical safety leaders in a tertiary hospital. Prospective evaluation through real-time observations of patient safety incidents. Medicine.

